# Exploring the Association between Complexity of Care, Medical Complexity, and Length of Stay in the Paediatric Setting Using a Nursing Minimum Data Set: A Study Protocol

**DOI:** 10.3390/nursrep14040213

**Published:** 2024-10-10

**Authors:** Manuele Cesare, Fabio D’Agostino, Antonello Cocchieri

**Affiliations:** 1 Center of Excellence for Nursing Scholarship (CECRI), Board of Nursing (OPI) of Rome, 00136 Rome, Italy; 2 Department of Medicine, Saint Camillus International University of Health Sciences, 00131 Rome, Italy; fabio.dagostino@unicamillus.org; 3 Section of Hygiene, Woman and Child Health and Public Health, Gemelli IRCCS University Hospital Foundation, Catholic University of the Sacred Heart, 00168 Rome, Italy; antonello.cocchieri@policlinicogemelli.it

**Keywords:** paediatrics, standardised nursing language, Nursing Minimum Data Set, complexity of care, nursing diagnoses, nursing actions, medical complexity, hospital length of stay, length of stay, Neonatal Paediatric Professional Assessment Instrument

## Abstract

Background/Objectives: The complexity of care requires systematic documentation to fully understand its relationship with medical complexity and its impact on patient outcomes. The Nursing Minimum Data Set (NMDS) plays a crucial role by capturing essential nursing data, enabling a detailed analysis of care and its impact on outcomes, such as length of stay (LOS). However, despite its potential, the use of NMDS in paediatric care remains limited. This study aims to explore the association between nursing and medical complexities and LOS in paediatric patients. Methods: A descriptive, retrospective, monocentric study will be conducted. The data will be collected through a nursing information system (Professional Assessment Instrument (PAI*ped*)) and the hospital discharge register of patients admitted to the paediatric department in 2022 in an Italian university hospital. Conclusions and Expected Results: The use of PAI*ped* will allow for the description of the complexity of care and enable an analysis of its relationship with medical complexity and LOS.

## 1. Introduction

The complexity of nursing care requires systematic documentation to effectively assess its relationship with medical complexity and its impact on patient outcomes [1,2]. Standardised nursing terminologies (SNTs) play a pivotal role in this process by offering a structured framework for recording and evaluating nursing practices, enhancing the visibility of nursing contributions to healthcare [3,4]. This structured approach not only justifies healthcare costs, but also informs policy development by allowing for comparisons with other disciplines [5,6,7].

One key method in assessing nursing care is the Nursing Minimum Data Set (NMDS), first introduced by Werley and Lang in the 1980s [8]. NMDS captures essential data, including patient demographics, nursing diagnoses (NDs), nursing interventions (NIs), nursing actions (NAs), and patient outcomes such as length of stay (LOS) [1,9]. This structured data collection enables a comprehensive evaluation of nursing care across healthcare settings and facilitates an understanding of how nursing and medical complexities interact [1,2,10].

The Nursing Dependency Index (NDI), based on NMDS data and determined by the number of NDs and corresponding NAs, serves as an indicator of care complexity [11]. The NDI, when integrated with medical data, provides valuable insights into the joint impact of nursing and medical conditions on patient outcomes [2,12]. However, despite its utility, the global use of NMDS-based data remains limited, indicating a need for further implementation and research [10].

In Italy, the Professional Assessment Instrument (PAI) was developed in 2012 to integrate the NMDS framework into electronic health records (EHRs) [13]. Research has demonstrated PAI’s effectiveness in various clinical settings, including oncology [9] and surgery [2], highlighting key connections between NDs and patient outcomes such as LOS. More recently, PAI has been adapted for use in neonatal and paediatric settings (PAI*ped*) [14]. However, research in paediatric care using PAI*ped* remains limited. With increasing medical complexity in paediatric populations driven by factors like chronic diseases and multimorbidity [15], there is a need to explore the relationship between medical complexity, nursing complexity, and health outcomes. Understanding this interaction is crucial for improving patient care and ensuring that both medical and nursing interventions are optimised to address the challenges posed by complex cases in paediatric healthcare [16,17].

While studies have indicated strong associations between NDs and outcomes like LOS [1,2,18], these relationships are underexplored in paediatric populations. Understanding the link between SNTs, medical data, and LOS in paediatrics could provide critical insights into factors influencing patient recovery, optimise care strategies, and identify key factors related to LOS. This knowledge could ultimately improve hospital cost control, patient care, and resource allocation [18,19]. Moreover, the standardised collection of nursing-generated data through PAI*ped*, combined with clinical details—such as medical diagnoses, diagnostic groupings, and risk assessments (e.g., fall and pressure ulcer risks) could offer a cleared picture of paediatric patient complexity. This comprehensive approach could enable a more effective analysis of how these factors collectively influence LOS [1,14].

### 1.1. Objective

The aim of this study is to explore the association between complexity of care, medical complexity, and length of stay (LOS) in the paediatric hospital setting using a NMDS.

#### Research Questions

(a) What is the prevalence of NDs, NIs, and NAs in paediatric patients?
(b) How do NDs, NIs, and NAs relate to clinical data, such as major or secondary medical diagnoses, Diagnosis Related Groups (DRGs), and clinical procedures?
(c) What is the relationship between NDs, NIs, NAs, and nursing measures for clinical risks (NMCRs) for the paediatric population, e.g., Humpty Dumpty Fall Scale (HDFS) [20] and Braden Q Scale [21]?
(d) How do NDs, NIs, and NAs influence LOS in paediatric patients when stratified by age group, medical diagnoses, and their groupings?
(e) How does the number of medical diagnoses influence the LOS, with NDs and NAs acting as mediators in this relationship?

## 2. Materials and Methods

### 2.1. Study Design

This is a quantitative, non-experimental, descriptive, retrospective, monocentric study. This protocol was registered with the Open Science Framework (OSF) under registration number DOI 10.17605/OSF.IO/JXYEQ on 29 July 2024.

### 2.2. Participants and Setting

This study will involve paediatric patients who were consecutively hospitalised at the A. Gemelli IRCCS University Hospital Foundation in Rome, Italy, between 1 January 2022 and 31 December 2022.

### 2.3. Inclusion Criteria

For this study protocol, participants must meet the following inclusion criteria: (1) patients hospitalised in the paediatric wards of the A. Gemelli IRCCS University Hospital Foundation, excluding those in the outpatient and day hospital units; (2) patients < 18 years old at the time of hospitalisation according to the criteria of Michelson and Neuman [22]; patients with LOS ≥ 2 days.

### 2.4. Variables

For each patient enrolled in the study, the following variables will be collected and analysed:

- Sociodemographic data, e.g., gender, age, education, rural urban classification.
- NDs, defined as «*a clinical judgment about the healthcare consumer’s response to actual and potential health conditions or needs*» [23]; ND is a granular condition based on the analysis and synthesis of the signs and symptoms assessed by the nurse. ND is the basis for the choice of NIs, which will lead to the achievement of the objectives for which the nurse is responsible (e.g., *Swallowing Impairment*, which is the «*inability to move food from mouth to stomach*») [24]. NDs are recorded according to the Clinical Care Classification (CCC) System (version 2.5) [14].
- Healthcare patterns, or 4 macro-categories which describe the holistic approach to patient care and categorise the care components (e.g., *Physiological*, *Psychological*, *Functional*, *Health behavioural*) [23].
- Care components, defined as a «*a cluster of elements that represent four unique patterns of clinical nursing practice: health behavioural, functional, physiological and psychological*»; they are represented by 21 groupings used to classify and code the CCC terminologies (CCC of NDs, CCC of NIs/NAs, CCC of nursing outcomes) (e.g., *Nutritional*, which is a *cluster of elements that involve the intake of food and nutrients*) [9,23].
- NIs, or a «*treatment, procedure, activity or service designed to achieve an outcome of a nursing or medical diagnosis for which the nurse is accountable*» (e.g., *Enteral Tube Care*, defined as the broad intervention «*performed to control the use of an enteral drainage tube*») [23]; since the NIs are granular concepts described at a general level, each one of them is associated with a set of NAs [24].
- NAs, which are specific behaviours put in place by nurses to perform the intervention in clinical practice [9]; they are described through the use of four action type qualifiers which focus on the specific action required for the implementation of each NI (e.g., monitor/assess, perform/care, teach/instruct, manage/refer) and represent different aspects of nursing care (e.g., *Perform Enteral Tube Insertion*). The use of NAs provides accurate metrics useful to assess the workload, but also to describe resources use and nursing costs associated with patient care [9,23,24].
- LOS, defined as the period elapsed between the patient’s hospital admission and discharge. A LOS that exceeds the 75th percentile of its total distribution will be defined as prolonged LOS (pLOS) [2].
- Diagnosis of illness or trauma (e.g., main medical diagnosis), other health problems (e.g., secondary medical diagnosis or comorbidities), causes of trauma, diagnostic and therapeutic procedures according to the international classification ICD-9-CM.
- DRGs, which are an international system that allows grouping patients with similar clinical characteristics and relatively homogeneous resource consumption [25].
- Major Diagnostic Categories (MDCs), which are grouping based on a criterion of clinical, anatomical, or etiological relevance and are formed by dividing all ICD-9-CM medical diagnoses into 25 specific diagnosis areas [2].
- NMCRs, e.g., Humpty Dumpty Fall Scale (HDFS), a validated tool that analyses 7 specific areas (Age, Gender, Diagnosis, Cognitive Impairments, Environmental Factors, Response to Surgery/Sedation/Anaesthesia, and Medication Usage) and identifies the presence of risk of falls in paediatric patients. The HDFS total score ranges between 7 and 23, and higher scores mean an increased fall risk [20]; Braden Q Scale, a predictive tool consisting of 7 items (Mobility, Activity, Sensory Perception, Moisture, Friction-Shear, Nutrition, Tissue Perfusion and Oxygenation) that can identify the risk of development of pressure injuries in paediatric patients). The Braden Q Scale total score ranges between 7 and 28, and higher scores indicate a decreased risk of skin integrity impairment [21]. In the study hospital, nurses use these scales to conduct clinical risk evaluations within the first 24 h from a patient’s hospital admission, according to Joint Commission International standards on quality of care and patient safety.

#### Framework of Causal Mediation between Medical Complexity, Complexity of Care, and Length of Stay

**Figure 1:**
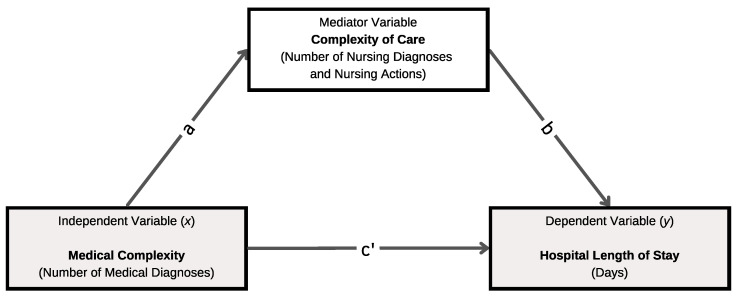
Hypothesis of a mediation analysis framework to understand the possible relationship between the independent variable, mediators, and outcome.

A mediation analysis framework (Figure 1) was hypothesised by the research group to outline the process by which the number of medical diagnoses (independent variable) could influence LOS (dependent variable) [18,26,27] specifically through the number of NDs and NAs (multiple mediators variables) [1,2].

More broadly, the theoretical justification of this framework, which is based on a literature analysis, supports the notion that the independent variable and mediators may have an impact on the dependent variable [28]. In particular, the medical complexity of patients, measured using the number of medical diagnoses [25], may have an impact on the patient complexity of care, as expressed by NDI and number of NAs [2,9,18]. According to our hypothesis, more numerous medical conditions may result in the identification of a major number of NDs and the need for more NAs [1,9], and this may have a direct effect on LOS [1], generating pLOS.

### 2.5. Source of Data

#### 2.5.1. Neonatal Paediatric Professional Assessment Instrument (PAI*ped*)

**Figure 2:**
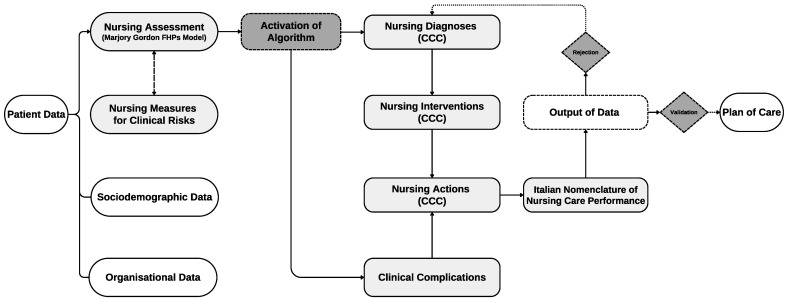
Content and structural architecture of PAI*ped*. Legend: FHPs, Functional Health Patterns; CCC, Clinical Care Classification.

The PAI*ped* is a Clinical Nursing Information System (CNIS) aimed at electronically recording hospital nursing care using a standardised nursing language, namely the CCC System (version 2.5) [14]. CCC is a framework developed across 4 levels (176 NDs, 201 NIs, 804 NAs, and 528 nursing outcomes), which have been tested for neonatal and paediatric care [23,24]. The PAI*ped* has an embedded clinical decision support system that supports nurses based on their assessment using Marjory Gordon’s Functional Health Patterns Model [3] in the choice of the appropriate NDs, Nis, and NAs [29]. However, the NDs or NIs and NAs identified using the clinical decision support system can be accepted or rejected by nurses, thus preserving the nurse’s decision-making autonomy [3]. Nurses can document their activities in the PAI*ped*, also using the Italian Nomenclature of Nursing Care Performance (INNCP) [9]. INNCP components are standard activities that provide comprehensive insights into the care process and enhance the understanding of CCC NAs through detailed descriptions. However, the PAI*ped* works in the background to map INNCP NAs to CCC NAs. This allows for a more comprehensive evaluation of the care provided by combining these two classification systems. Nurses are also supported in identifying specific clinical complications related to each assessment performed. Overall, PAI*ped* includes 251 clinical complications related to neurosurgical, cardiovascular, pulmonary, and internal disorders [14]. For each clinical complication suggested and selected by nurses, the PAI*ped* provides a list of recommended NAs that nurses can validate and implement in clinical practice to reduce the risk of unfavourable patient outcomes. Nurses can also document NMCRs using specific validated tools embedded in the PAI*ped*, which are suitable for paediatric patients. Actually, the PAI*ped* is an essential component of the study hospital’s EHR. Due to their utilisation of PAI*ped* since 2016, the nurses at the study hospital can be regarded as proficient users of the system [14]. The content and structural architecture of PAI*ped* is shown in Figure 2.

**Figure 3:**
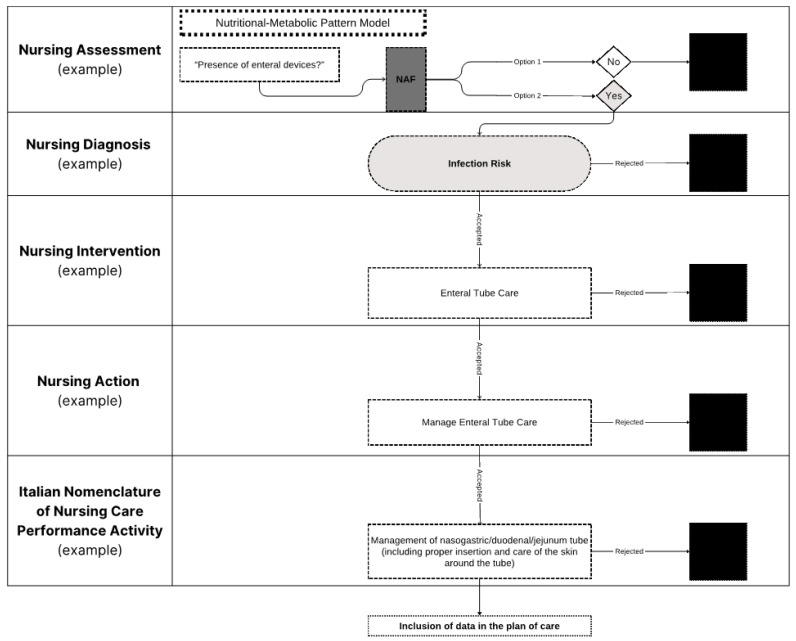
Example of NAF activation on PAI*ped*. Legend: NAF, Nursing Assessment Form. Note: the black square indicates data that are rejected by the nurse and not included in the plan of care.

An example of how the PAI*ped* algorithm (called the Nursing Assessment Form—NAF) [29] operates, generating NDs, NIs, NAs, and INNCP components that must be validated by nurses, is illustrated in Figure 3.

#### 2.5.2. Hospital Discharge Register (HDR)

The HDR is the standard tool for collecting information on every patient discharged from public and private institutions in Italy [1]. HDR uses the international ICD-9-CM classification system to code both diagnostic and therapeutic procedures. This classification system organises diseases and traumatisms into groups based on defined criteria. The HDR collects information including personal details of the patient (e.g., age, gender, residence, level of education), hospitalisation characteristics (e.g., admission regime, discharge modality), and clinical characteristics (e.g., principal and secondary medical diagnosis, diagnostic or therapeutic procedures) [30].

### 2.6. Data Extraction

Data extraction from the hospital’s EHR will involve the Information and Communication Technologies (ICT) team systematically querying the EHRs to extract relevant patient information and linking different datasets or sources to ensure comprehensive data collection. This process will be executed using specialised software tools designed to interface with the EHR system, ensuring that data are accurately retrieved and organised according to predefined criteria. The extracted data will be anonymised to protect patient privacy before being compiled into a dataset for detailed analysis. This meticulous process guarantees that the data are both comprehensive and secure, facilitating robust and reliable research outcomes.

### 2.7. Data Analysis

The data will be analysed using descriptive statistical methods. NDs, NIs, and NAs, medical diagnoses, DRGs, and clinical procedures will be described with absolute frequencies and percentages. Continuous variables, such as LOS and the average number of NDs, will be described with the mean and standard deviation if the distributions are normal, or otherwise with the median and interquartile range. For the study of significant associations between the number of NDs, NIs, Nas, and clinical variables (e.g., number of medical diagnoses, also considering their groupings and LOS), a parametric or non-parametric correlation will be performed depending on the variables taken into account. Multiple correlation matrices will be included to measure the maximum degree of linear relationships between multiple independent variables and a single dependent variable, providing a more comprehensive understanding of complex interdependencies. For the study of significant relationships between medical complexity (e.g., number of medical diagnoses), complexity of care, and LOS, appropriate strategies for mediation analysis (e.g., Baron and Kenny mediation method [31]) will be used to estimate the effects of intermediate variables (or mediators, e.g., NDs and NAs) on the relationship between the number of medical diagnoses and LOS. To generate more robust evidence, all variables will be systematically coded. For example, medical diagnoses, NDs, NAs, and LOS will be categorised appropriately, and statistical tests, such as the Chi-square test, will be applied to assess associations between categorical variables and to support the mediation analysis. We will conduct both aggregated and stratified analyses based on age and disease condition to accurately assess the variability in patient characteristics and LOS. This approach will enable us to identify and compare the effects of different developmental stages and nursing and medical conditions on hospitalisation metrics, ensuring that age-specific and pathology-specific nuances are thoroughly examined. Proper analytical strategies will be used to reduce confounding, include or eliminate possible interaction terms, and address missing data [32]. Given the number of statistical tests being conducted, the critical *p*-value will be adjusted using an appropriate multiple comparisons correction to control for Type I errors end ensure the validity of the results. Associations with a *p*-value < 0.05 will be considered statistically significant. Statistical analysis will be performed using the software IBM^®^ SPSS^®^ Statistics for Mac OS, Ver. 29 (Armonk, NY, USA: IBM Corp.).

### 2.8. Ethics Statement

The study protocol was approved by the Ethics Committee of the Catholic University of the Sacred Heart in Rome, Italy (Protocol no. 0012915/24, ID 6752, approved 16 May 2024). All paediatric data will be collected after obtaining written informed consent (via a cover letter) from the patients’ parents or legal guardians to participate in the study. Additional informed consent will be required from nurses involved in clinical documentation, who will record the data in EHRs. Informed consent will be obtained before data collection, following a thorough explanation of the study’s purpose and methodology. Parents, legal guardians, and nurses will be free to opt out of the study whenever they express their will. The dataset will be anonymised by the data warehouse, and it will not be possible to identify the study participants under any circumstances. This study will be conducted in accordance with the principles of good clinical practice, the Code of Ethics of the World Medical Association, the Declaration of Helsinki, and current regulations. This study will not interfere with clinical practice. The research group will ensure that any relevant amendments to the protocol that occur during the study are forwarded to the Ethics Committee by the principal investigator.

## 3. Discussion

Our study protocol will be crucial for detailing the prevalence of NDs, NIs, NAs, and NMCRs in paediatric care, and for analysing how these factors relate to patient sociodemographic characteristics and LOS. Past research has attempted to pursue similar objectives [1,2,18], but did not consider paediatric patients and the set of variables we propose in this protocol, such as NAs and NMCRs. This approach would provide a deeper understanding of the complexity of care in this study population.

Determining the complexity of care is a critical issue for nursing research and, in general, for health systems. Understanding the complexity of care is essential for evaluating patient characteristics, their dependence on nursing care, the impact of nursing care on patient outcomes, and for planning and managing nursing resources [1,2]. However, only a few approaches have been developed to characterise complexity of care. As several studies have shown, there is considerable ambiguity and measurement challenges related to the complexity of care due the lack of a distinct and shared definition [33,34]. Consequently, some care settings, such as paediatrics, seem to be underexplored in relation to this dimension.

To the best of our knowledge, this study will be the first in the literature aimed at demonstrating the complexity of care for paediatric patients using reliable and reproducible criteria, specifically those suggested by the NMDS [10], and incorporating standardised nursing data [4]. Our study protocol aims to fill this gap in the literature by adopting a measurement strategy based on the latest advances in the field. We chose to measure the complexity of care by considering the NDI, NMCRs, and the number of NAs performed by nurses in clinical practice to address the NDs identified after the nursing assessment [9]. As several authors have shown, a higher number of NDs and NAs can create a “trigger” that determines higher levels of complexity of care [1,2]. As a result, the use of standardised NDs and NAs could serve as a useful and accurate indicator for this purpose. This methodology would also allow us to share, replicate, and compare our results worldwide across different populations and settings of care.

The entire research process will be based on the analysis of standardised nursing-generated data. Since this research approach has only been applied to adult patients thus far, including the use of the PAI system, our study may knowledge about the complexity of care in the paediatric field, which remains unexplored. The implementation of a data collection strategy based on the extraction of standardised data acquired through a CNIS that supports nurses in the selection of NDs and NAs through a validated algorithm will add core value to our study. The collected data will enable future comparisons across different populations, settings, and geographic areas, as suggested by NMDS principles [10]. Moreover, to improve the overall description of patient complexity, our study protocol will also include the collection of data related to risk assessments conducted by nurses during their practice (e.g., NMCRs). These data will also be analysed in relation to other variables and key health outcomes (e.g., LOS). Data will be used to test several research hypotheses (e.g., the relationships between NDs, NAs, and NMCRs), and any additional factors that emerge during the study period will also be investigated. Our findings will be crucial for assessing the effectiveness of NIs and NAs in relation to LOS and will encourage future research into conducting cost analyses based on SNTs [4,35,36].

Moreover, implementing this study protocol will be essential for exploring the connections between complexity of care and other health dimensions, such as medical complexity. Although the literature has established that these two factors are independent and distinct knowledge base and scope [37,38], they have never been systematically compared to scientifically determine any potential similarities or differences. Specifically, data extracted from a NMDS have not been used as distinct dimensions for comparison with medical complexity, particularly in paediatric populations. This study will be the first to illustrate how the complexity of care is distributed across different medical diagnoses and their groupings. This research is crucial due to the urgent need to describe various dimensions of patient care complexity and their significant implications for both research and practice [1].

The study of the association between nursing variables (NDs, NAs, and NMCRs) and LOS will introduce a novel key element to the research. To date, only a limited number of studies involving adult patients have examined these relationships using data from SNT-based CNISs [1,2,9]. Our research design will provide a new perspective on how these variables relate to LOS, which affects the quality, safety, and costs of care [19], particularly in the paediatric setting [26,27]. These analyses will have significant implications for evaluating the sustainability of health systems from a public health perspective.

Despite its innovative nature, this study protocol may have some limitations. An inherent limitation is the use of a retrospective study design, which relies on data documented in the study hospital’s EHR rather than data specifically gathered for research purposes at the time of entry. Additionally, since this study design will be implemented after the outcomes of interest have already occurred, this may affect the generalisability of the results [39]. Potential biases in data extraction from the hospital’s EHR might include inaccuracies in EHR entries, human errors, and insufficient anonymisation. To mitigate these biases, we will implement rigorous validation processes, use multiple data sources for cross-checking, and adhere to strict anonymisation protocols and standardised extraction criteria.

Another limitation could be the adoption of a monocentric design. Such a design may involve a limited patient population, potentially affecting the statistical power and robustness of the results. To address this, we will clearly define the patient demographics and clinical practices of the study hospital. This transparency will help evaluate the relevance of our findings to other settings and guide future multicentric studies. Since the study is conducted in a single centre, results may not fully represent broader populations or diverse clinical settings, which could affect the generalisability of the findings. We will perform thorough statistical analyses and account for the sample size in interpreting our results. Additionally, we will also emphasise the need for replication in larger multicentric studies to validate and extend our findings across different settings and populations.

However, this research also has several strengths, including its innovative goals and the robust methodology planned to explore our hypotheses. While a monocentric design has been noted as a limitation, it also offers several advantages that justify its use in this study. Specifically, it ensures uniformity in data collection procedures and diagnostic criteria, provides consistent data, and maintains controlled conditions, leading to reliable results. The experience and expertise of the study hospital staff with PAI*ped* can further enhance data collection quality, potentially yielding more-robust findings. These findings could guide future studies, inform multicentric research, and influence clinical practices.

We will use rigorous statistical methods to analyse the data and include confidence intervals to account for the sample size. Additionally, we will clearly state the limitations related to the sample size to provide context for the findings. We will thoroughly describe the characteristics of the study hospital and the patient population to contextualise our results. The context-specific nature of the findings will be discussed, and we will suggest directions for future research to explore the applicability of the results in different settings.

The consecutive recruitment of paediatric patients over a full calendar year will improve the generalisability of the results, minimising the impact of seasonal fluctuations. This approach will offer a precise and comprehensive definition of the complexity of care in this setting. As a result, we will be able to relate this dimension to medical data, primary outcomes, and organisational outcomes of care, providing the scientific community with new and updated evidence. This study will also facilitate the formulation of new research hypotheses regarding other potential and hidden factors associated with the complexity of care.

## 4. Relevance for Clinical Practice, Policy, and Research

The implementation of this study protocol will be pivotal in enhancing clinical practice, research, and health policy. By applying NMDS principles to gather standardised nursing data, this study aims to characterise paediatric nursing care. The PAI*ped* will use a systematic approach to collect standardised nursing data [4,14], presenting these findings in nursing research for the first time. By integrating nursing data with HDR information, the study will offer valuable insights into care processes, uncover connections with patient clinical and social information, and investigate how nursing variables impact LOS. Additionally, this study can serve as a roadmap for similar future research by offering a robust methodological framework. This framework will facilitate the timely identification of patient needs in paediatric populations, address nursing health education promotion, and evaluate the impact of nursing care on patient outcomes [35,40]. Our structured approach will facilitate a detailed analysis, bridging research gaps and deepening the understanding of patient complexity, ultimately guiding health policies and improving patient care.

## 5. Conclusions and Expected Results

This study will explore the association between complexity of care, medical complexity, and LOS in paediatric patients, providing critical insights into how nursing and medical complexities interact and influence LOS. By utilising the PAI*ped* system, we will comprehensively assess nursing care, including clinical risk evaluations based on NMCRs, which will allow for a detailed understanding of care complexity within the paediatric population. We believe that the results of this research may have significant applications in clinical practice, with key impacts on public health. The use of PAI*ped* will facilitate a detailed description of nursing care in the paediatric setting, including assessments performed by nurses based on NMCRs.

The approach outlined in this protocol will allow us to analyse patient characteristics and compare LOS across various developmental stages, ensuring accurate consideration of age and clinical condition-related differences. This will help contextualise our results and provide a clearer understanding of how medical and nursing complexity of care varies by age and conditions. Further research on age-specific subgroups will be recommended to refine our findings and develop targeted interventions for diverse paediatric populations.

The data generated from both nursing and medical sources will enhance the understanding of the complexity of care, offering opportunities to hypothesise predictive models for selected outcomes. The investigations outlined in this study protocol will address different dimensions of nursing and medical care, with results having significant implications at multiple levels, including clinical, organisational, managerial, political, and economic areas.

## Data Availability

The dataset generated and analysed during the conduction of the study presented in this protocol will be not publicly available due to participants privacy, confidentiality and ethical restrictions. The data collected from the implementation of this study will be available upon reasoned request to the corresponding author.

## Public Involvement Statement

No public involvement in any aspect of this research.

## Guidelines and Standards Statement

This manuscript was drafted following the STROBE guidelines [41] for observational research.
